# Artificial intelligence-enabled social robots for facilitating social interactions in patients with dementia: a systematic review

**DOI:** 10.3389/fpsyg.2026.1825127

**Published:** 2026-07-31

**Authors:** Junjie Wang, Jing Shen, Jiayi Wang, Ruopeng An

**Affiliations:** 1 School of Sports and Health Promotion, Dalian University of Technology, Dalian, China; 2 Department of Physical Education, China University of Geosciences, Beijing, China; 3 School of Applied Science, Taiyuan University of Science and Technology, Taiyuan, China; 4 Silver School of Social Work, New York University, New York, NY, United States

**Keywords:** artificial intelligence, dementia, social interactions, social robots, systematic review

## Abstract

**Background:**

The global rise in dementia, closely linked to aging populations, necessitates innovative interventions to address care challenges. Artificial Intelligence-Enabled Social Robots (AI-ESRs) present a promising approach by leveraging multi-modal communication to enhance social engagement and alleviate isolation. However, evidence regarding their efficacy remains inconsistent and fragmented. This systematic review evaluates the effectiveness of AI-ESRs in enhancing social interactions among people with dementia, examines factors contributing to outcome variability, and provides recommendations for optimizing their integration into care models.

**Methodology:**

A comprehensive search was conducted across five electronic databases (PubMed, Web of Science Core Collection, EBSCO, Scopus, and Cochrane Library) for peer-reviewed articles published up to May 2026. Inclusion criteria encompassed studies addressing AI-ESRs interventions in dementia care, following the Preferred Reporting Items for Systematic Reviews and Meta-Analyses (PRISMA) guidelines. Data extraction was standardized to ensure consistency across selected studies.

**Results:**

16 studies met the inclusion criteria, comprising seven randomized controlled trials (RCTs) and 9 pre-post studies. 15 studies demonstrated the potential of AI-ESRs to significantly enhance multiple dimensions of social interactions. These included verbal and non-verbal communication, the frequency and quality of interactions with caregivers, peers, and robotic systems, as well as behavioral indicators (e.g., smiling and laughter) and participation in activities. One study reported no statistically significant differences between intervention and control groups (*p* = 0.18) but noted a marginal improvement in communication scores.

**Conclusions:**

AI-ESRs show promise in facilitating social interactions among patients with dementia, with their effectiveness influenced by participant characteristics, intervention design, and contextual factors. Further research is warranted to explore long-term outcomes, tailor interventions to individual needs, and address ethical and implementation challenges, thereby advancing the integration of AI-ESRs into dementia care.

## 1 Introduction

The global demographic landscape is undergoing profound transformations, marked by a rapid and unprecedented increase in the aging population. According to the World Health Organization (WHO), the number of individuals aged 60 years and older is projected to more than double to 2.1 billion by 2050, while the population aged 80 years and older is expected to triple to 426 million by the same year (WHO, 2024). This demographic shift is accompanied by a corresponding rise in age-related conditions, with dementia emerging as one of the most pressing public health challenges (Grande et al., 2020).

Dementia, closely associated with aging, is a syndrome characterized by progressive cognitive decline and impaired daily functioning, encompassing a range of diseases that affect memory, cognition, and activities of daily living (Mok et al., 2024). Alzheimer's disease is the most common form of dementia, contributing to approximately 60–80% of cases (Clifford et al., 2024). Alarmingly, approximately one person develops dementia globally every 3 s (Andriella et al., 2020). The WHO estimates that 55 million people worldwide are living with dementia, with about 10 million new cases diagnosed annually. In 2019, global economic costs attributable to dementia reached US$ 1.3 trillion, with approximately 50% of these costs arising from informal caregivers, including family members and close friends, who provide an average of 5 h of care and supervision daily (WHO, 2023). These figures symbolize formidable challenges to healthcare systems, social services, and economies worldwide.

In response to the rising prevalence and impact of dementia, a variety of pharmacological and non-pharmacological interventions have been explored. Pharmacological treatments primarily target the cholinergic system, neuroprotection, and inflammation, but have shown limited efficacy in altering disease progression (Dang et al., 2024). In contrast, non-pharmacological interventions have shown considerable promise in improving patients' quality of life while causing fewer adverse effects (Park and Lee, 2024; Ying et al., 2024). These non-pharmacological interventions, including physical exercise (Hu et al., 2024), music therapy (Cho et al., 2024), cognitive rehabilitation (Brunelle-Hamann et al., 2015), and staff education (Leone et al., 2013), have proven effective in mitigating psychotic symptoms such as delusions and hallucinations. However, a notable gap exists in these approaches: they often fail to comprehensively address social interactions, a critical determinant of cognitive health and emotional wellbeing in patients with dementia (Arai et al., 2021).

Emerging research highlights the potential of artificial intelligence (AI)-enabled social robots (AI-ESRs) to enhance social interactions in dementia care. AI-ESRs are intelligent, physically embodied agents that represent a significant departure from conventional, fixed-logic predecessors. By leveraging machine learning, computer vision, and natural language processing (NLP), these AI-driven systems possess the capacity to perceive, interpret, and dynamically respond to human affective cues and behaviors in real time, moving beyond the constraints of pre-programmed, static automation (Kalotra, 2023; Lee, 2023). These robots employ multi-modal communication—including text, voice, and images—to simulate human-like interactions and foster user engagement. For instance, while a conventional robotic toy may play a recorded sound upon touch, an AI-ESRs can analyze a user's facial expression via computer vision and adjust its conversational tone through NLP to offer empathetic responses. Such capabilities may be instrumental in establishing trust and therapeutic alliances, encouraging adherence to treatment regimens, and reducing social isolation (Anisha et al., 2024; Rettinger et al., 2024). Despite these prospects, significant barriers to adopting AI-ESRs in dementia care persist. Ethical considerations, technical challenges, and the high costs of development and deployment continue to hinder their widespread implementation (Asl et al., 2023; Bradwell et al., 2020).

Moreover, while some studies report positive outcomes—ranging from improved communication (Kiuchi et al., 2023) and enhanced social interaction (Lu et al., 2021) to improved emotional wellbeing (Hsieh et al., 2023) and reduced loneliness (Lim, 2023)—the evidence remains inconsistent. Variability in its effectiveness can be attributed to heterogeneity in study designs, differences in the types of robots employed, and variations in the individual characteristics of patients with dementia (Hirt et al., 2021). For example, patients at different stages of dementia may exhibit varying levels of engagement with robotic interventions, and cognitive impairments may limit meaningful interactions for some individuals. Furthermore, the novelty of AI-ESRs raises concerns about the long-term sustainability of their benefits, as the enduring impacts of these interventions remain underexplored.

Therefore, this systematic review aims to examine the role of AI-ESRs in facilitating social interactions among people with dementia. By analyzing existing evidence, it seeks to identify key factors influencing the effectiveness of these interventions, highlight gaps in the literature, and provide recommendations to optimize the integration of AI-ESRs in dementia care.

## 2 Methods

This systematic review followed the Cochrane Handbook for Systematic Reviews of Interventions, and its reporting complies with the Preferred Reporting Items for Systematic Reviews and Meta-Analyses (PRISMA) guidelines.

### 2.1 Study selection criteria

Studies that met the following criteria were included in the review: (1) Study design: controlled intervention; (2) Participants: older adults with dementia; (3) Exposure: AI-enabled social robot; (4) Outcomes: social interaction (e.g., verbal, quasi-linguistic, or behavioral interactions); (5) Time window of search: peer-reviewed articles published from the inception of a bibliographic database until July 2024; the search was subsequently updated in May 2026 to include newly published eligible studies; and (6) Language: English. Studies that met any of the following criteria were excluded from the review: (1) conference proceedings, letters to editor, dissertations, or case reports; (2) review articles; (3) non-intervention studies; (4) non-human studies; and (5) pre-print articles.

### 2.2 Search strategy

A keyword search was performed across five electronic bibliographic databases: PubMed, Web of Science Core Collection, EBSCO, Scopus, and Cochrane Library. The search algorithm included all possible combinations of the keywords from the following two groups: (1) “dementia” and (2) “social robot”. All keywords in the PubMed were searched with the “[Title/Abstract]” tag, which is processed using Automatic Term Mapping (NLM, 2024). The search function TS = Topic was used in Web of Science, EBSCO, Scopus, and Cochrane Library, which launches a search for topic terms in the fields of title and abstract (Clarivate, 2020; Cochrane library, 2024; EBSCO, 2019; Elsevier, 2024). The search algorithm spanning these five electronic bibliographic databases is reported in Appendix 1. Titles and abstracts of the articles identified through the keyword search were screened. Potentially relevant articles were retrieved to evaluate the full text. Two reviewers, JJW and JYW, independently screened titles and abstracts and identified potentially relevant articles. Discrepancies were resolved through face-to-face discussion between JJW, JYW and RA.

A comprehensive reference search was conducted using both backward and forward methodologies on full-text articles that met the study selection criteria identified through the initial keyword search. Articles discovered through these reference searches were subsequently screened and evaluated using the same criteria. This iterative process of reference searching continued until no additional relevant articles were discovered.

### 2.3 Data extraction

A standardized data extraction form was utilized to systematically collect each study's key methodological and outcome variables. The variables extracted included authors, publication year, country/region, study design, sample size, participant sex and age, disease status, setting, AI robot type, intervention components, intervention duration, data collection method, evaluation time points, outcome measure, and estimated intervention effects.

A meta-analysis was not conducted due to significant heterogeneity in outcome measures and methodologies across the included studies. The lack of consistent quantitative results and variations in study design precluded the aggregation of data for meaningful statistical synthesis.

### 2.4 Study quality assessment

Study quality was assessed using the National Institutes of Health Quality Assessment of Controlled Intervention Studies. The questions on the assessment tool were designed to help reviewers focus on the key concepts for evaluating a study's internal validity. For each criterion, a score of 1 was assigned if “yes” was the response, whereas a score of 0 was assigned otherwise (i.e., an answer of “no,” “not applicable,” “not reported,” or “cannot determine”). A study-specific global score, ranging from 0 to 14, was calculated by summing up scores across all criteria. The study quality assessment helped measure the strength of scientific evidence but was not used to determine the inclusion of studies.

## 3 Results

### 3.1 Literature search

**Figure 1:**
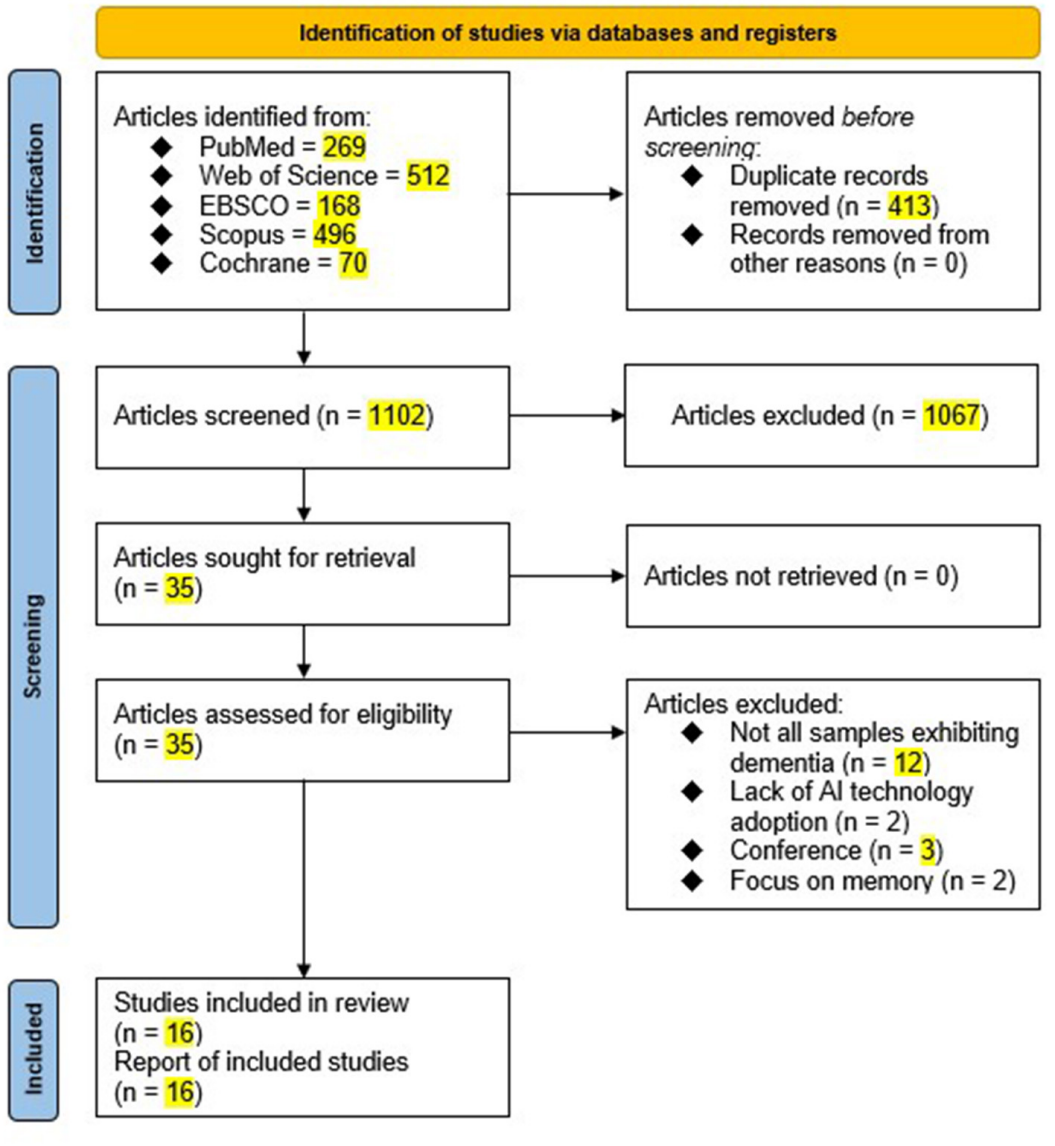
Study selection flowchart.

Figure 1 presents the PRISMA flow diagram. We identified 1,515 articles through keyword search, including 269 from PubMed, 512 from Web of Science, 168 from EBSCO, 496 from Scopus, and 70 from Cochrane Library. After removing 413 duplicate records, 1,102 unique articles were retained for screening. These articles underwent title and abstract screening, thereby excluding 1,067 articles that did not meet the inclusion criteria. Subsequently, 35 articles were selected for full-text review. Following a comprehensive evaluation of the full-text articles, 19 studies were excluded for various reasons, such as the absence of dementia among all samples (*n* = 12), lack of AI technology adoption (*n* = 2), being conference proceedings (*n* = 3), and focusing on memory rather than social interactions (*n* = 2). In total, 16 studies met the selection criteria and were included in the review (Bradwell et al., 2022; Chen et al., 2022a,b; Dinesen et al., 2022; Inoue et al., 2022; Joranson et al., 2016; Liang et al., 2017; Mazuz and Yamazaki, 2026; Moyle et al., 2019, 2017; Obayashi and Masuyama, 2020; Obayashi et al., 2020; Perdomo-Delgado et al., 2025; Sung et al., 2015; Tulsulkar et al., 2021; Whelan et al., 2020).

### 3.2 Study characteristics

A total of 16 studies published between 2015 and 2026 were included in this review. The studies were conducted across multiple country/regions, including Japan, Taiwan, Australia, Denmark, Norway, New Zealand, Singapore, Spain, Israel, Ireland, and the UK. 7 studies employed randomized controlled trials (RCTs) designs, while nine utilized pre–post intervention designs.

Sample sizes ranged from 2 to 415 participants, with most studies including fewer than 100 individuals. Participants were older adults with varying degrees of dementia recruited from nursing homes, long-term care facilities, day care centers, and community settings. The AI-ESRs evaluated fell into two primary categories: animaloid models (with “Paro” being the most frequently utilized) and humanoid models (such as Palro, Nadine, MARIO, and RoBoHoN). Detailed study characteristics are presented in Table 1.

**Table 1:** Characteristics of the studies included in the review.

ID	First Author, year	Country /region	Design	Sample size	Sex	Age (years)	Disease status	Setting	AI-robot type
1	Bradwell et al., 2022	UK	RCTs	63 IG: 26 CG: 37	Female: 49 Male: 14	Mean: Int: 86.92 ± 7.29 Con: 88.05 ± 8.48	Dementia severity score (0–54): IG: 27.30 ± 14.89 CG: 28.51 ± 19.93	Nursing, residential	JFA (Animal)
2	Chen et al., 2022a	Taiwan	Pre-post	24	Female: 16 Male: 9	Mean: 82.60 ± 5.60 Range: 68–91	MMSE score: mean: 25.60 ± 1.70	Nursing homes	Paro (Animal)
3	Chen et al., 2022b	Taiwan	RCTs	52	IG: Female: 65.4% CG: Female: 42.3%	Mean: IG: 81.81 ± 8.54 CG: 79.08 ± 7.71 Range: 65– 96	MMSE score: IG: 22.04 ± 2.88 CG: 21.50 ± 2.50	Long-term care facilities	Paro (Animal)
4	Dinesen et al., 2022	Denmark	Pre-post	42Individual session: 12 Group session: 30	Individual session: Female: 11 Male: 1 Group session: Female: 22 Male: 8	Individual session: Mean: 83 Range:67– 92 Group session: Mean: 84 Range: 66–96	Individual session: Mean years with dementia: 2 Group session: Mean years with dementia: 3.5	Nursing homes	LOVOT (Animal)
5	Inoue et al., 2022	Japan	Pre-post	77	Female: 68 Male: 9	Mean: 86.70 ± 6.20 Range: 67–97	53 Alzheimer's disease 9 vascular dementia 15 other dementia	Nursing homes, participants' homes	Palro (Humanoid)
6	Joranson et al., 2016	Norway	Cluster RCTs	23	Female: 16 Male: 7	Range: 62– 92 Mean: 84.70 ± 7.00	Mild to severe dementia	Nursing homes	Paro (Animal)
7	Liang et al., 2017	New Zealand	RCTs	24 IG: 13 CG: 11	Female: 16 Male: 8	Range: 67–98	Dementia	Nursing homes, participants' homes	Paro (Animal)
8	Moyle et al., 2017	Australia	Cluster RCTs	415 Paro group: 138 Toy group: 140 Usual care group: 137	Paro group: Female: 101 Male: 37 Toy group: Female: 114 Male: 26 Usual care group: Female: 99 Male: 38	Paro group: Mean: 84.00 ± 8.40 Toy group:Mean: 86.00 ± 7.60 Usual care group: Mean: 85.00 ± 7.10	Dementia	Long-term care facilities	Paro (Animal)
9	Mazuz and Yamazaki, 2026	Israel	RCTs	53 IG1: 7 IG2: 12 CG: 34	NA	Range: 65-92	Dementia	Day care center	RoBoHoN (Humanoid)
10	Moyle et al., 2019	Australia	Cluster RCTs	20 Paro group: 10 Plush toy group: 10	Paro group: Female: 7 Male: 3 Plush toy group: Female: 8 Male:2	Paro group: Range: 20–65 Plush toy group: Range: 20–65	Dementia	Long-term care facilities	Paro (Animal)
11	Obayashi et al., 2020	Japan	Pre-post	2	Female	Patient 1: 104 Patient 2: 87	Mild dementia	Nursing home	Sota (Animal)
12	Obayashi and Masuyama, 2020	Japan	Pre-post	78	Female: 68 Male: 10	Mean: 86.50 ± 7.70	Dementia	Nursing home	Cota (Type: NA) Palro (Humanoid)
13	Perdomo-Delgado et al., 2025	Spain	Pre-post	13	Female: 13	Mean: 81.60	Mild dementia	Hospital setting	JFA (Animal)
14	Sung et al., 2015	Taiwan	Pre-post	12	Female: 3 Male: 9	Mean: 77.25 ± 6.70	Years of diagnoses with medical illness: 1.33 ± 0.78	Long-term care facilities	Paro (Animal)
15	Tulsulkar et al., 2021	Singapore	Pre-post	14	NA	Older	Cognitive impaired	Nursing home	Nadine (Humanoid)
16	Whelan et al., 2020	Ireland	Pre-post	10	Female: 7 Male: 3	83.00 ± 10.10	2 mild dementia 6 moderate dementia 2 severe dementia	Nursing home	MARIO (Humanoid)

RCTs, randomized clinical trials; IG, intervention group; CG, control group; MMSE, Mini-mental state examination; JFA, Joy for All, NA, not available.

Intervention protocols and outcome assessments varied across studies. Most interventions spanned between 4 and 12 weeks, with session frequencies typically ranging from 1 to 3 times per week (15 to 60 min per session). Outcomes were assessed using standardized instruments, behavioral observations, and video or audio recordings. While all studies adopted pre–post assessments, six included additional longitudinal follow-up time points. Details of intervention characteristics, outcome measures, and key findings are summarized in Table 2.

**Table 2:** Intervention characteristics and main findings in the studies included in the review.

ID	Intervention components	Intervention duration (weeks)	Data collection method	Evaluation time points	Outcomes	Estimated intervention effects
1	The intervention was the implementation of JfA cat and dog robots, which were provided to the intervention group care homes to use as they felt appropriate.	16	Scale (Holden Communication Scale)	Baseline, week 16	Communication	There was no statistically significant difference in communication issues between the control and intervention groups from baseline to follow-up between the control and intervention groups (1.41 ± 6.00 vs. 0.65 ± 7.54, *p* = 0.18). The communication scores increased slightly in the intervention group (16.58 ± 11.85 vs. 17.23 ± 15.33).
2	Participants engaged in 24 sessions of 60-min interactions with Paro over 8 weeks, at a frequency of three sessions per week.	8	Semi-structured interview	NA	Interactions with Robot and people	The study demonstrated that interaction with Paro could enhance social engagement among participants. Specifically, Paro facilitated increased verbal and nonverbal communication, stimulated social interactions with peers, and promoted a sense of social connection.
3	Participants in the experimental group received an individual, non-facilitated, 60-min PARO intervention 3 times per week for 8-weeks with a total of 1,440 min. The control group received treatment as usual activities, including individual drawing, painting, or singing.	8	Scale (engagement of a person with dementia scale)	Baseline, week 4, 8, and 12	Social interaction	Participants in the PARO intervention group demonstrated significantly greater social interaction than the control group. This effect was significant for the group, time, and interaction (*p* < 0.001), suggesting that the PARO intervention effectively enhanced social engagement in individuals with dementia.
4	Participants engaged in individualized LOVOT sessions (20-30 min, twice weekly for 4 weeks) and group sessions (30-45 min, twice weekly for 12 weeks)	Individual session: 4; Group session: 12	Semi-structured interview	Individual session: baseline, week 4; group session: baseline, week 12	Social interaction	Robot LOVOT had positive effects, opened up communication, and facilitated interpersonal interaction in both individual and group sessions.
5	The participants interacted with PALRO for 30 min leisure program, including self-introduction, ice breaker/small talk, light exercise, quiz and game, seated exercise with music, short stories, taking a photo, and closing remarks.	30 mins	Behavioral observation	Before (30 mins), during (30 mins), after (30 mins)	Engagement with object	A PALRO-based leisure program elicits a good response from participants, especially in engagement with the object (e.g., staring at PALRO).
6	Paro sessions were conducted twice weekly for 10 weeks during the intervention period, totaling 20 sessions. Facilitated by an activity leader, sessions focused on spontaneous interactions between participants and the Paro robot.	12	Video recordings	week 2, 10	Behavioral variables (e.g., conversation with object)	The results revealed statistically significant differences in two behavioral variables. “Smile/laughter toward other participants” increased from 0.8% to 1.5% (−0.7, *p* = 0.011). Conversely, “Conversation with Paro on the lap” decreased from 9.0% to 6.7% (2.3, *p* = 0.014).
7	Participants in the experimental group received 2-3 weekly, 30-min unstructured sessions with Paro at a daycare center and were also provided with Paro for home use for 6 weeks. The control group engaged in standard daily activities, including quizzes, exercise, bingo, music, and word games.	12	Behavioral observation	Baseline, week 6 and 12	Social interaction	Paro significantly enhanced staff communication in daycare centers (*p* = 0.042), with a mean score of 46.9 ± 26.5 compared to the control group's 25.5 ± 24.3.
8	Upon introduction to the social robot (RoBoHoN), participants utilized an unstructured, bottom-up “free association” format, communicating spontaneously whatever came to mind.	Session 1: 25 mins, Session 2: 35 mins	Behavioral observation, interviews	Baseline, end of intervention	Physical, visual, verbal, and non-verbal interactions	The social robot prompted an immediate, pronounced increase in active participation and communicative engagement, fostering both peer-to-peer and human-robot interactions within the group setting.
9	Participants were randomly assigned to one of three groups: PARO intervention, plush toy intervention, or usual care. Those in the PARO and plush toy groups received three 15-min sessions per week for 10 weeks. The PARO group interacted with a PARO therapeutic robot, while the plush toy group interacted with a plush toy. The usual care group received standard care.	10	Video recordings	Baseline, week 1, 5, and 10	Verbal and visual engagement	The Paro intervention group were significantly more verbally (3.61, 95% CI: 6.40–0.81, *P* = 0.011) and visually engaged (13.06, 95% CI: 17.05–9.06, *P* < 0.0001) with the object (Paro) than the plush toy group after 10 weeks.
10	Participants underwent a 10-week PARO intervention, consisting of 3 15-min unfacilitated sessions per week. Participants were free to interact with PARO as desired.	10	Video recordings	Baseline, week 1, 5, and 10	Social engagement	Relatives of long-term care residents with dementia reported positive perceptions of the Paro as a tool for facilitating communication.
11	The Sota robot was strategically placed in designated areas to facilitate real-time interaction with participants over a four-day period.	4	Voice recording	Baseline, day 1, 2, 3, and 4	Number and frequency of conversation with other	Compared with the case before the introduction of Sota robot, the number of conversations with care workers increased from a mean of 11.3–14.5, and conversations with residents increased significantly from 1.5 to 10.3 (*P* < 0.05). Conversations with the robot increased from 4 times on the 1st day to 8 times on the fourth day.
12	Participants received either Robot Cota or Robot Palro for an eight-week intervention. Robot Cota was programmed to provide task-oriented reminders, while Robot Palro was designed for social interaction and recreational engagement. Both robots were situated on participants' bedside tables and programmed for regular interactions during designated times or activities.	8	Behavioral observation	Baseline, week 8	Conversation activities	Participants exhibited enhanced conversational abilities, including speech fluency and interpersonal interactions with peers.
13	Participants received two sessions per week, with each individual session lasting approximately 45 to 50 min. Each session adhered to a structured format: Welcome and orientation, Free interaction, Guided activities, and Brief reflection.	8	Behavioral observation, semi-structured Interviews	Baseline, week 8	Social Interaction	Objective field notes revealed clear emotional engagement via affectionate smiling, rhythmic petting, and increased verbalization both to the robot and peers. This interaction yielded an extended “spillover” socialization effect beyond clinical sessions, prompting spontaneous conversations with non-enrolled day-hospital residents.
14	Participants received a group robot assisted therapy using a seal-like robot pet for 30 min twice a week for 4 weeks. The participants took turn and were encouraged to interact with Paro by talking, touching, and stroking.	4	Scale (assessment of communication and interaction skills, Activity participation scale)	Baseline, week 4	Communication and interaction skills, activity participation	The result showed that participants' communication and interaction skills (59.81 ± 8.95 vs. 73.58 ± 6.36, *z* = −2.94, *P* = 0.003) and activity participation (8.55 ± 2.84 vs. 11.5 ± 2.50, *z* = −2.66, *P* = 0.008) were significantly improved after receiving 4-week robot-assisted therapy.
15	Participants engaged in 29 one-on-one interactions with Nadine, with the help of care staff. Following these sessions, Nadine was autonomously active for 3 h each morning for 6 days. During interactions, residents could initiate conversations, with Nadine initiating dialogue when prolonged inactivity was observed.	NA	Scale (Menorah park engagement scale), video recording	Baseline, end of intervention	Level of engagement	Robot Nadine's interactions resulted in a notable increase in resident engagement quality and duration. As measured by the Menorah Park Engagement Scale, participants' engagement time rose from below 50% to over 50% of the session.
16	A MARIO social robot was deployed in a 5-week intervention with individuals diagnosed with dementia. Participants engaged in up to 12 sessions, each lasting approximately 24 min, 3 times per week. The intervention protocol involved MARIO introducing itself by name and using verbal and visual cues to stimulate user engagement.	5	Semi-structured interviews, observational measure of engagement	Baseline, week 5	Engagement levels, interactions with the robot	MARIO robot intervention significantly increased positive engagement by 17% while reducing negative engagement by 10%.

NA: not available.

Overall, the evidence suggests that AI-ESRs interventions can improve both verbal and nonverbal social interactions among people with dementia. Reported benefits included increased communicative engagement, interaction frequency, visual attention, smiling, and laughter across a range of social settings.

### 3.3 Study quality assessment

The studies included in this review exhibited variations in methodological quality, as summarized in Table 3. The average quality score was 9.4 out of 14, with scores ranging from 7 to 13. Only six of the 14 studies explicitly identified themselves as RCTs (Bradwell et al., 2022; Chen et al., 2022b; Joranson et al., 2016; Liang et al., 2017; Moyle et al., 2019, 2017). Furthermore, the adequacy of randomization was confirmed in four studies (Bradwell et al., 2022; Chen et al., 2022b; Joranson et al., 2016; Liang et al., 2017; Moyle et al., 2019), where random assignment methods such as random number generation were employed to reduce selection bias. Blinding, a standard procedure to minimize bias, was less frequently applied. Only two studies (Chen et al., 2022b; Moyle et al., 2019) blinded participants and providers, while another two (Dinesen et al., 2022; Moyle et al., 2019) blinded outcome assessors. The limited use of blinding suggests a potential risk of bias, as knowledge of treatment assignments could influence participants' behavior and outcomes assessment. Except for one study (Chen et al., 2022a), baseline comparability of groups on key characteristics was enforced across all studies, indicating that participants were balanced regarding essential traits such as demographics, risk factors, and co-morbid conditions. All studies maintained low overall and differential drop-out rates. Adherence to intervention protocols was high, and other interventions were avoided or kept consistent across groups. Outcome assessments were conducted using valid and reliable measures, consistently applied to all participants. At the same time, only 4 studies (Chen et al., 2022b; Inoue et al., 2022; Liang et al., 2017; Moyle et al., 2017) reported that their sample size was sufficiently large to detect statistically significant differences in outcomes with at least 80% power. All studies adhered to pre-specified outcome reporting and used intention-to-treat analysis.

**Table 3:** Quality assessment of the studies included in the review.

Criteria	Study id															
	1	2	3	4	5	6	7	8	9	10	11	12	13	14	15	16
1. Was the study described as randomized, a randomized trial, a randomized clinical trial, or an RCT?	1	0	1	0	0	1	1	0	1	1	0	0	0	0	0	0
2. Was the method of randomization adequate (i.e., use of randomly generated assignment)?	1	0	1	0	0	1	1	0	1	0	0	0	0	0	0	0
3. Was the treatment allocation concealed (so that assignments could not be predicted)?	1	0	1	0	0	1	1	0	1	0	0	0	0	0	0	0
4. Were study participants and providers blinded to treatment group assignment?	0	0	1	0	0	0	0	0	1	0	0	0	0	0	0	0
5. Were the people assessing the outcomes blinded to the participants' group assignments?	0	0	0	1	0	0	0	0	1	0	0	0	0	0	0	0
6. Were the groups similar at baseline on important characteristics that could affect outcomes (e.g., demographics, risk factors, co-morbid conditions)?	1	0	1	1	1	1	1	1	1	1	1	1	1	1	1	1
7. Was the overall drop-out rate from the study at endpoint 20% or lower of the number allocated to treatment?	1	1	1	1	1	1	1	1	1	1	1	1	1	1	1	1
8. Was the differential drop-out rate (between treatment groups) at endpoint 15 percentage points or lower?	1	1	1	1	1	1	1	1	1	1	1	1	1	1	1	1
9. Was there high adherence to the intervention protocols for each treatment group?	1	1	1	1	1	1	1	1	1	1	1	1	1	1	1	1
10. Were other interventions avoided or similar in the groups (e.g., similar background treatments)?	1	1	1	1	1	1	1	1	1	1	1	1	1	1	1	1
11. Were outcomes assessed using valid and reliable measures, implemented consistently across all study participants?	1	1	1	1	1	1	1	1	1	1	1	1	1	1	1	1
12. Did the authors report that the sample size was sufficiently large to be able to detect a difference in the main outcome between groups with at least 80% power?	0	0	1	0	1	0	1	0	0	1	0	0	0	0	0	0
13. Were outcomes reported or subgroups analyzed prespecified (i.e., identified before analyses were conducted)?	1	1	1	1	1	1	1	1	1	1	1	1	1	1	1	1
14. Were all randomized participants analyzed in the group to which they were originally assigned, i.e., did they use an intention-to-treat analysis?	1	1	1	1	1	1	1	1	1	1	1	1	1	1	1	1
**Total Score**	11	7	13	9	9	11	12	8	13	10	8	8	8	8	8	8

## 4 Discussion

This systematic review evaluated the effectiveness of AI-ESRs in facilitating social interactions among individuals with dementia. The findings underscore the potential of AI-ESRs to significantly improve multiple dimensions of social interactions, including verbal and non-verbal communication, the frequency and quality of interactions with staff, peers, and robotic systems, as well as observable behavioral indicators (i.e., smile/laughter toward others), and participation in activities. However, the outcomes demonstrate considerable variability across studies, largely due to the differences in methodological rigor, sample characteristics, and intervention protocols. These findings offer critical insights into the efficacy of AI-ESRs, highlighting both their promise and the challenges associated with their implementation in dementia care.

### 4.1 Efficacy of AI-ESRs in enhancing social interactions

This review reports predominantly positive outcomes across studies, with 13 out of 14 investigations reporting significant improvements in various aspects of social engagement. This consistent pattern suggests that AI-ESRs can effectively facilitate social interactions among individuals with dementia, with positive effects observed across multiple domains.

First, verbal and non-verbal communication demonstrated notable improvements. Studies consistently reported improvements in both the quantity and quality of verbal exchanges, as evidenced by increased conversation frequency and duration. These findings corroborate prior research indicating that social robotic interventions can stimulate communicative behaviors in individuals with cognitive impairments (Lin et al., 2022; Rodríguez-Domínguez et al., 2024). The improvement in non-verbal communication, particularly through increased eye contact, facial expressions, and gestural responses, suggests that AI-ESRs can effectively engage patients across multiple communicative channels. This aligns with previous research suggesting that participants in socially assistive robot interventions often establish eye contact and respond appropriately to the robots' requests (Lin et al., 2022; Rettinger et al., 2024). Second, the studies revealed enhanced social engagement patterns, both with the robots and, more importantly, with human counterparts. This documented increase in resident-to-resident interactions (e.g., from 1.5 to 10.3 interactions in Obayashi's study) suggests that AI-ESRs may serve as social catalysts, facilitating broader social engagement beyond the immediate human-robot interaction. This finding aligns with prior research indicating that such robots can enhance the quality of communication between care recipients and their caregivers (Dosso et al., 2023). This “spillover effect” could be particularly valuable in mitigating social isolation in institutional settings. Third, the findings indicate improved behavioral indicators of social engagement, including increased smiling and laughter, suggesting enhanced emotional wellbeing during social interactions. These outcomes align with earlier studies reporting positive emotional responses to socially assistive robots among individuals with dementia (Otaka et al., 2024). The emotional dimension of these interactions is particularly critical, as positive affective experiences during social engagements can reinforce social participation and contribute to sustained behavioral improvements over time.

### 4.2 Comparative effectiveness of AI-robot types

The review highlighted distinct patterns of effectiveness between animaloid and humanoid robots in therapeutic contexts. Animaloid robots, particularly PARO, consistently demonstrated positive outcomes across multiple studies. This consistency may be attributed to their calming appearance and ability to elicit nurturing behaviors, while avoiding unrealistic expectations related to complex social interactions. The simplified social dynamics of animaloid robots appear particularly well-suited for individuals with moderate to severe dementia, as noted in prior research (Traeger et al., 2020). In contrast, humanoid robots demonstrated potential in facilitating structured interactions, particularly in domains such as cognitive stimulation and guided conversations. Moro et al. observed that participants expressed a preference for humanoid robots, treating them as companions, perceiving them as more useful, and showing a greater willingness to integrate them into social or private areas of their homes (Moro et al., 2019). However, evidence supporting the effectiveness of humanoid robots remains comparatively limited. Moreover, their impact appears to depend heavily on the cognitive status of the participants, thereby necessitating further investigation.

### 4.3 Theoretical mechanisms underlying AI-ESRs effects on social interaction

The observed effects of AI-ESRs on social interaction among individuals with dementia can be understood through several complementary neurocognitive and psychosocial mechanisms. (1) Reduced cognitive load: Social withdrawal among people living with dementia is partly intensified by the substantial neurocognitive burden associated with conventional interpersonal communication (Porcelli et al., 2019). Effective human interaction largely relies on rapid conversational turn allocation, continuous interpretation of nuanced and often ambiguous social signals, and the real-time generation of contextually appropriate responses (Holler et al., 2015). AI-ESRs can alleviate these interactional demands by functioning as predictable, cognitively accessible social partners. Their capacity to operate without requiring complex bidirectional linguistic exchanges, coupled with a high tolerance for delayed responses and periods of silence, creates a less demanding communicative environment. These characteristics lower the cognitive resources necessary for initiating and maintaining social participation, thereby facilitating social engagement among individuals with dementia. (2) Predictable and non-threatening interaction patterns: Rather than functioning as a static exchange of information, human communication is an adaptive process influenced by emotional states, social relationships, and environmental factors (Solomon et al., 2021). In contrast, AI-ESRs offer stable, and highly predictable responses. Such consistency may reduce uncertainty-related stress, anxiety, and behavioral resistance commonly observed in dementia care. From the Lawton's ecological model of adaptation and old age, adaptive functioning is optimized when environmental demands are aligned with an individual's remaining competencies (Izal et al., 2005). AI-ESRs may therefore facilitate engagement by providing a communicative environment calibrated to the user's cognitive capabilities. (3) Affective stimulation (particularly for animaloid robots): Animaloid robotic platforms-most notably PARO-simultaneously engage tactile, auditory, and visual processing modalities, delivering integrated multimodal sensory-affective stimulation. Synthesizing paradigms from cognitive reminiscence theory and attachment behavior frameworks, these pet-like entities are capable of triggering deeply ingrained caregiving heuristics, attachment dynamics, and emotionally meaningful memories (Fearon and Roisman, 2017).

### 4.4 Methodological considerations

Several methodological limitations warrant consideration when interpreting the findings. First, variability in study designs: the reviewed studies included randomized controlled trials (RCTs) and pre-post intervention designs, with RCTs generally exhibiting stronger internal validity. Nonetheless, a significant proportion of studies did not employ randomization or blinding procedures, thereby introducing potential biases. Only six studies explicitly identified themselves as RCTs, and of these, only four adequately detailed their randomization methods. This inconsistency raises concerns surrounding the reliability of certain findings and underscores the need for more rigorous trial designs in future research. Second, sample characteristics: the sample sizes across studies were relatively small, with only one study exceeding 100 participants (Moyle et al., 2017). Such limited sample sizes may reduce statistical power and generalizability. Moreover, most studies recruited participants with varying stages of dementia, which may have influenced the observed outcomes. For example, individuals with mild cognitive impairment may engage more effectively with robots than those with advanced dementia, highlighting the need to tailor interventions to individual needs. Third, robot type: animal-like robots (e.g., PARO, JFA) predominantly elicit affective and non-verbal responses, likely via biomimetic features activating emotional memory. Humanoid robots (e.g., MARIO, Nadine) may engage higher-order cognitive and verbal functions, potentially attenuating communicative willingness (Fan et al., 2025). This distinction likely accounts for a portion of the cross-study variability in verbal vs. non-verbal outcome profiles. Finally, intervention protocols: intervention duration and frequency varied considerably, ranging from single 30-min sessions to multi-week programs. While longer interventions often demonstrated sustained improvements in social engagement, shorter interventions yielded more modest effects. Additionally, the lack of standardized intervention protocols further complicates comparisons across studies. For example, some studies employed structured activities guided by the robot, while others allowed spontaneous interaction, leading to divergent outcomes.

### 4.5 Contextual determinants and implementation

The therapeutic efficacy of AI-ESRs is fundamentally moderated by contextual determinants, specifically clinical or residential setting, cognitive stage, and organizational infrastructure. In acute hospital environments, AI-ESRs function as “humanizing agents” that mitigate the anxiety and sensory deprivation inherent in restrictive clinical spaces by providing immediate psychosocial comfort (Shi et al., 2025). Within long-term care facilities-where the majority of empirical evidence is situated-the presence of structured group sessions, caregiver supervision, and peer social dynamics shifts the robot's role. Here, AI-ESRs operate primarily as “social bridges,” facilitating resident-to-resident interactions that counteract social isolation (Obayashi and Masuyama, 2020). This collective format effectively amplifies communicative opportunities through shared social dynamics. Conversely, evidence from home-based deployments reveals distinct interaction paradigms characterized by the absence of peers. In these unstructured environments, the AI-ESRs assumes the role of the principal social interaction partner rather than merely facilitating engagement. Consequently, continued use relies substantially on caregiver support, whereas factors such as technology acceptance, usability, and maintenance requirements become significant determinants of long-term adoption (Nam and Park, 2025). In addition, intervention success is strictly contingent upon “technological-organizational fit”; high staff workload and inadequate digital infrastructure—such as unreliable Wi-Fi—often transform potential AI benefits into perceived labor burdens, thereby stifling adoption and reducing implementation fidelity (Lee et al., 2025; Wong et al., 2024). The “how and why” behind these variations are rooted in the alignment between the robot's AI capabilities and the user's functional needs. For individuals in early dementia stages, robots facilitate social signaling and cognitive engagement. Recent evidence highlights significant improvements in verbal communication, particularly through enhanced speech production and conversational quality (Chen et al., 2022a). For those with advanced impairment, the therapeutic mechanism shifts to AI-driven affective computing—leveraging computer vision and NLP to interpret non-verbal cues—which provides sensory regulation and emotional bonding when linguistic capacity is diminished. Consequently, AI-ESRs have been associated with greater visual attention, prolonged eye contact, increased frequency of smiling, enhanced affective vocalizations, and other forms of socio-emotional expressiveness (Joranson et al., 2016; Perdomo-Delgado et al., 2025). Ultimately, without robust institutional support to activate the robot's adaptive intelligence, these systems fail to yield meaningful improvements in social health and wellbeing (Melkas et al., 2020; Papadopoulos et al., 2020).

### 4.6 Ethical reflexivity and practice implications

The integration of AI-ESRs into dementia care necessitates a nuanced analysis of five pivotal ethical domains. First, inequitable access highlights how current deployment models often prioritize English-speaking residents, potentially marginalizing those from diverse linguistic backgrounds or advanced cognitive stages. Second, consent barriers underscore the inadequacy of static institutional protocols; we advocate for a “relational ethics” approach that interprets non-verbal cues as ongoing assent. Third, residents' privacy is increasingly compromised by high-frequency data collection—via computer vision and NLP—which risks transforming therapeutic environments into sites of digital surveillance. Fourth, the substitution of human care represents a critical moral tension, where robots must be strictly positioned as assistive adjuncts rather than replacements for essential human empathy and touch. Finally, the risk of infantilization must be mitigated by designing interactions that honor the life histories of older adults, preventing the reduction of social robots to mere “toys” that undermine user dignity (Dosso et al., 2025; Hung et al., 2025; Wong et al., 2024). Addressing these domains is essential for ensuring that AI-ESRs serve as instruments of genuine empowerment rather than social exclusion.

### 4.7 Limitations of current evidence

Several limitations in the existing evidence warrant further attention. First, the lack of blinding and randomization in many studies undermines the robustness of the findings. Second, the reliance on diverse outcome measures and inconsistent evaluation time points could further complicate data synthesis and interpretation. Third, the novelty of AI-ESRs means that their long-term effects remain poorly understood. Most studies focused primarily on short-term outcomes, leaving critical doubts about the sustainability of AI-ESRs' benefits.

### 4.8 Recommendations for future research

In-lieu of the above-mentioned limitations, we recommend that future studies prioritize the following aspects of AI-ESRs research: (1) standardizing methodology: developing standardized protocols for intervention duration, frequency, and evaluation metrics will enable more reliable comparisons and meta-analyses; (2) larger and more diverse samples: recruiting larger and more diverse participant cohorts will enhance the generalizability of findings and enable subgroup analyses to identify patient-specific factors influencing outcomes; (3) longitudinal studies: investigating the long-term effects of AI-ESRs on social interactions, cognitive health, and emotional wellbeing that will provide valuable insights toward their sustained impact; (4) ethical and economic considerations: future research should explore strategies that address ethical concerns and reduce costs, thereby facilitating equitable access to these technologies; (5) comparative effectiveness research: comparing AI-ESRs with other non-pharmacological interventions will help delineate their unique contributions to dementia care; (6) standardized effect sizes: utilizing Cohen's *d* or partial η^2^ to quantify the practical magnitude of intervention gains, rather than solely relying on statistically significant pre-to-post changes.

## 5 Conclusions

AI-ESRs demonstrate significant potential to enhance social interactions among individuals with dementia, addressing a critical gap in current care strategies. However, their effectiveness is influenced by a complex interplay of factors, such as individual patient characteristics (e.g., stage of dementia), robot type (e.g., humanoid features), and intervention parameters (e.g., duration, intensity, and frequency). Despite promising outcomes, substantial gaps remain in our understanding of the long-term sustainability and scalability of these interventions. Future research should prioritize longitudinal studies that assess long-term impacts, refine intervention frameworks to accommodate individual variability, and explore adaptive mechanisms to optimize engagement. Concurrently, ethical considerations—including patient autonomy, data privacy, and informed consent—must be rigorously addressed to ensure responsible deployment. Additionally, overcoming technical and financial barriers is essential to facilitate broader implementation. By systematically addressing these challenges, AI-ESRs can be effectively integrated into dementia care paradigms, contributing to improved patient outcomes, and advancing the field of geriatric healthcare innovation.
