# Supplementary material for: Artificial intelligence-enabled social robots for facilitating social interactions in patients with dementia: a systematic review
Source: Front Psychol. 2026 Jul 31;17:1825127. doi: 10.3389/fpsyg.2026.1825127 (PMC13475613; doi:10.3389/fpsyg.2026.1825127)
Supplement: Supplementary file 1 [file Supplementary_file_1.docx]

**Appendix 1. Search strategy**

(1) **PubMed**

(“Dementia”[MeSH Terms] OR “Dementia”[Title/Abstract] OR (“Alzheimer Disease”[MeSH Terms] OR “Alzheimer Disease”[Title/Abstract]) OR “Cognitive Decline”[Title/Abstract] OR “Neurodegenerative Disease”[Title/Abstract] OR “mild cognitive impairment”[Title/Abstract]) AND (“social robotic”[Title/Abstract] OR “social robotics”[Title/Abstract] OR “social robot”[Title/Abstract] OR “social bot”[Title/Abstract] OR “social bots”[Title/Abstract] OR “social robots”[Title/Abstract] OR “assistive robot”[Title/Abstract] OR “assistive robots”[Title/Abstract] OR “companion robot”[Title/Abstract] OR “companion robots”[Title/Abstract] OR “personal robot”[Title/Abstract] OR “personal robots”[Title/Abstract] OR “interactive robot”[Title/Abstract] OR “interactive robots”[Title/Abstract] OR “robotic companion”[Title/Abstract] OR “robotic companions”[Title/Abstract] OR “human-robot interaction”[Title/Abstract] OR “human-computer interaction”[Title/Abstract] OR “humanoid robot”[Title/Abstract] OR “humanoid robots”[Title/Abstract])

(2) **Web of Science**

“Dementia” OR “Alzheimer Disease” OR “Cognitive Decline” OR “Neurodegenerative Disease” OR “mild cognitive impairment” (Topic) and “social robotic” OR “social robotics” OR “social robot” OR “social bot” OR “social bots” OR “social robots” OR “assistive robot” OR “assistive robots” OR “companion robot” OR “companion robots” OR “personal robot” OR “personal robots” OR “interactive robot” OR “interactive robots” OR “robotic companion” OR “robotic companions” OR “human-robot interaction” OR “human-computer interaction” OR “humanoid robot” OR “humanoid robots” (Topic) and Article (Document Types)

(3) **EBSCO**

Subject terms (“Dementia” OR “Alzheimer Disease” OR “Cognitive Decline” OR “Neurodegenerative Disease” OR “mild cognitive impairment”) AND (“social robotic” OR “social robotics” OR “social robot” OR “social bot” OR “social bots” OR “social robots” OR “assistive robot” OR “assistive robots” OR “companion robot” OR “companion robots” OR “personal robot” OR “personal robots” OR “interactive robot” OR “interactive robots” OR “robotic companion” OR “robotic companions” OR “human-robot interaction” OR “human-computer interaction” OR “humanoid robot” OR “humanoid robots”) and Article (Document Types)

(4) **Scopus**

( TITLE-ABS-KEY ( ( “social robotic” OR “social robotics” OR “social robot” OR “social bot” OR “social bots” OR “social robots” OR “assistive robot” OR “assistive robots” OR “companion robot” OR “companion robots” OR “personal robot” OR “personal robots” OR “interactive robot” OR “interactive robots” OR “robotic companion” OR “robotic companions” OR “human-robot interaction” OR “human-computer interaction” OR “humanoid robot” OR “humanoid robots” ) ) AND TITLE-ABS-KEY ( ( “Dementia” OR “Alzheimer Disease” OR “Cognitive Decline” OR “Neurodegenerative Disease” OR “mild cognitive impairment” ) ) ) AND ( LIMIT-TO ( DOCTYPE , “ar” ) )

(5) **Cochrane Library**

(“Dementia” OR “Alzheimer Disease” OR “Cognitive Decline” OR “Neurodegenerative Disease” OR “mild cognitive impairment”) in Title Abstract Keyword AND (“social robotic” OR “social robotics” OR “social robot” OR “social bot” OR “social bots” OR “social robots” OR “assistive robot” OR “assistive robots” OR “companion robot” OR “companion robots” OR “personal robot” OR “personal robots” OR “interactive robot” OR “interactive robots” OR “robotic companion” OR “robotic companions” OR “human-robot interaction” OR “human-computer interaction” OR “humanoid robot” OR “humanoid robots”) in Title Abstract Keyword
